# Differential significance of Seminal Cell-Free DNA Levels, Oxidative Stress, and Sperm Characteristic between Infertile Men with Non-obstructive and obstructive Azoospermia

**DOI:** 10.5935/1518-0557.20250007

**Published:** 2025

**Authors:** Davoud Javidmehr, Farzaneh Fesahat, Fatemeh Hassani, Ali Reza Talebi, Abdolhossein Shahverdi

**Affiliations:** 1 Department of Reproductive Biology,Faculty of Basic Sciences and Advanced Technologies in Medicine,Royan Institute, ACECR, Tehran, Iran; 2 Reproductive Immunology Research Center, Shahid Sadoughi University of Medical Sciences, Yazd, Iran; 3 Department of Embryology, Reproductive Biomedicine Research Center, Royan Institute for Reproductive Biomedicine, ACECR, Tehran, Iran; 4 Department of Biology & Anatomical Sciences, Shahid Sadoughi University of Medical Sciences, Yazd, Iran

**Keywords:** azoospermia, seminal plasma, cell-free DNA, oxidative stress

## Abstract

**Objective:**

The levels of cfDNA in the semen samples of infertile men and its relationship with the level of oxidative stress, antioxidant capacity and lipid peroxidation were investigated.

**Methods:**

Total 100 semen samples were obtained from infertile men with abnormal sperm parameters (oligoasthenospermia (n=10), oligoteratozoospermia (n=10), oligoasthenoteratozoospermia (n=10), and non-obstructive azoospermia (n=50)) and normozoospermic men. cfDNA was extracted and quantified by qPCR. Different markers related to the oxidant and antioxidant status as well as oxidative stress were measured in the seminal plasma samples of the study groups.

**Results:**

cfDNAs content in the oligoasthenoteratozoospermia group was significantly higher than those in the controls. A significant decrease in cfDNAs was observed in the azoospermia group compared to the controls. There was a significant increase in malondialdehyde and other oxidant related markers in all patient groups rather than the normal individuals. In contrast, seminal plasma samples of all patients with abnormal semen parameters showed a significant reduction in the levels of factors associated with antioxidant capacity compared to the controls.

**Conclusions:**

This study highlights the direct link between elevated cfDNA levels, oxidative stress, and impaired sperm parameters in men with azoospermia and oligospermia. Current data underscore the potential competence of cfDNA and oxidative stress as diagnostic tools to classify the severity of male infertility and abnormalities related to semen parameters. Our findings emphasize the importance of antioxidant therapy as an efficient strategy to reduce oxidative damage, enhance sperm quality, and improve reproductive outcomes for male partners of couples with infertility.

## INTRODUCTION

Infertility, defined as the inability to conceive after 12 months of regular unprotected intercourse, affects approximately 15% of couples globally, with male factors contributing to around 35% of these cases. The spectrum of male infertility includes various conditions such as oligozoospermia, asthenospermia, azoospermia-including Non-obstructive (NOA) or obstructive (OA), teratozoospermia, and varicocele (Sharma et al., 2021).

While traditional semen analysis remains the cornerstone of male infertility diagnostics, its limitations in definitive diagnosis necessitate the exploration of novel biomarkers and technologies (Bieniek et al., 2016). Conventional methods used to diagnose male infertility, are only successful in detecting the most severe types of male factor infertility, such as NOA, which affects around 10% to 15% of infertile men (Aitken et al., 2020). This limitation has led to interest in innovative biomarkers, such as seminal cell-free DNA (Cf-DNA) and indications of oxidative stress (Llavanera et al., 2022).

The documentation regarding the presence of free nucleic acids in seminal plasma is limited (Wu et al., 2013). Li et al. (2009) demonstrated that levels of seminal Cf-DNA were markedly elevated in azoospermic patients compared to people without any abnormalities in sperm. Elsewhere, it has been reported that increased levels of seminal Cf-DNA are associated with abnormalities in sperm motility and morphology (Costa et al., 2017). A correlation was demonstrated between the quantity of free mitochondria in semen and sperm characteristics (Chen et al., 2018).

Reactive oxygen species (ROS), linked to male infertility, have been documented since the 1940s. Early studies showed that oxidative stress impairs sperm function, highlighting the harmful effects of naturally produced hydrogen peroxide (H2O2) on sperm metabolism and motility (MacLeod, 1943). These factors highlight the need for comprehensive analysis beyond traditional semen parameters (Dehghanpour et al., 2017; Tabibnejad et al., 2019). Men with NOA may have increased levels of ROS, which can worsen DNA damage and hinder the function of any existing spermatozoa, further complicating their already limited reproductive potential. On the other hand, oxidative stress levels in OA may be relatively reduced or regulated differently because of the intact production of sperm and the lack of sperm cell arrest (Aitken et al., 2014).

This study seeks to investigate the varying importance of seminal CF-DNA levels, oxidative stress, and sperm characteristics in the diagnosis and understanding of the varied causes of non-obstructive and obstructive azoospermia. Through the incorporation of up-to-date research findings and observations from clinical practice, our aim is to shed light on the intricate interaction of these factors in male infertility and the possible consequences they may have on treatment approaches.

## MATERIAL AND METHODS

### Study Design

This study included a total of 100 semen samples, divided into a control group (Normospermia) and several infertile groups. The Normospermia group consisted of 20 individuals who were confirmed to have normal semen parameters prior to the study, based on standard semen analysis as recommended by the World Health Organization (WHO) (2021). All participants signed an informed consent form before sample collection. This study was approved by the Ethics Committee of Royan Research Institute with code IR.ACECR.ROYAN.REC.1401.042.

The infertile groups consisted of 80 samples with abnormal semen parameters and were further categorized into the following subgroups based on their specific infertility diagnoses: 10 men with Oligoasthenospermia (OA), 10 men with Oligoteratozoospermia (OT), 10 men with Oligoasthenoteratozoospermia (OAT), and 50 men with non-obstructive azoospermia (AZO) (Abdulrazak, 2021; Imani et al., 2023).

The inclusion criteria for the patient groups were a diagnosis of infertility and a body mass index (BMI) below 30. For the control group, men with normal semen parameters and no diagnosed infertility were selected. Exclusion criteria for all participants included obstructive azoospermia (e.g., varicocele), pyospermia (defined as more than one million white blood cells per milliliter of semen), tobacco use, infectious diseases, diabetes, and alcohol consumption. This selection ensured that the study focused on the biological markers related specifically to non-obstructive infertility.

### Sample collection and semen analysis

the semen samples were collected participants referred to Yazd reproductive sciences institute. The consent form was signed by all participants before entering the study.

Samples were obtained via masturbation following a period of 2-5 days of refraining from sexual activity. During the liquefaction process, the samples were kept at a temperature of 37°C for 20 minutes. The assessment of sperm characteristics was conducted based on the 2021 standards established by the World Health Organization (WHO). To evaluate the movement of sperm, a total of 100 spermatozoa were seen under a phase-contrast microscope with a magnification of ×400. The proportions of motile, non-motile, and immotile sperm cells were examined according to the WHO guidelines (World Health Organization, 2021). The semen was centrifuged at 400xg for 10 minutes to separate the supernatant, which was then transferred to a new tube and centrifuged at 12,000xg for 10 minutes to minimize cellular DNA contamination. The resulting seminal plasma was stored at -80°C for PCR analysis. Each sample was analyzed in triplicate for reliability.

### Cell-Free DNA Quantification

To extract nucleic acids, 2 ml of thawed seminal plasma was processed using the ARTOUN Cell-Free DNA Isolation Kit (Farasou Gene Fanavar Co, Iran), following the manufacturer’s protocol. Initially, 2 ml of plasma was mixed with 2 ml of CFL buffer, 30 µl of Super Carrier, and 200 µl of Proteinase K solution, agitated, and incubated at 60°C for 30 minutes. Then, 4 ml of CFB buffer was added and vortexed. Spin Columns with Column Extenders were rinsed with 600 µl of CFW1 buffer, 750 µl of CFW2 buffer, and 750 µl of ethanol, with vacuum applied after each rinse. The columns were then centrifuged at 13000 rpm for 2 minutes. Next, 50 µl of CFE buffer was added to the columns, incubated for 1 minute, and centrifuged at 13000 rpm for 30 seconds.

For quantitative real-time PCR, 5 µl of cfDNA was mixed with 20 µl of PCR LightCycler® 480 SYBR Green I Master (Roche Diagnostics), 2.5 mM MgCl2, and 0.5 mM of each forward (5’-AGATTTGGACCTGCGAGCG-3’) and reverse (5’-GAAGCCGGGGCAACTCAC-3’) RNaseP primers. The amplification was performed using a Light Cycler 480 II (Roche Diagnostics) with 35 cycles of 95°C for 10 seconds, 59°C for 20 seconds, and 72°C for 15 seconds, followed by a 5-minute elongation at 72°C. The Ct values were compared to a standard curve to determine the cycle threshold, and the cfDNA concentration was averaged from three observations.

### Antioxidant enzyme and lipid peroxidation assay

The samples were centrifuged at 3000 RPM for 10 minutes and the supernatant was separated. The biochemical parameters, such as total antioxidant capacity (TAC), total organic carbon (TOC), glutathione peroxidase (GPX), and malondialdehyde (MDA), were quantified (using ZellBio GmbH, Germany) following the manufacturer’s protocols. In the TAC assay, a volume of 10 µL of serum was mixed with reaction buffer and chromogen reagent. The absorbance of the mixture was then measured at a wavelength of 450 nm. The total organic carbon (TOC) concentration was evaluated by adding 50 µL of serum to the reaction mixture and measuring the absorbance at 600 nm. The GPX activity was evaluated by combining 20 µL of serum with a reaction mixture including glutathione and NADPH, and measuring the reduction in absorbance at 340 nm. MDA levels were quantified by incubating 50 µL of serum with a mixture of thiobarbituric acid (TBA) reagent and chromogen solution and then measuring the absorbance at 535 nm. As indicated, the measurements were performed using either a spectrophotometer or a microplate reader.

### Statistical Analysis

Statistical analysis was performed with GraphPad Prism software version 9 (GraphPad Inc. in San Diego, CA, United States). First, the patient group was compared with the control group, and then the patient group was divided into four subgroups. Student’s t-test was used to compare two groups and one-way ANOVA was used to compare different patient groups with the control group and Tukey’s post-test. A p<0.05 was considered as a significant level.

## RESULTS

### Age, body weight and semen analysis results

Table 1 provides a summary of the age, body weight, and semen analysis results for both the patients and control groups. There were no statistically significant differences in age and body mass index (BMI) among all groups. Significant differences in sperm parameters were observed between the control group (Normospermia) and the various infertile groups. There were no statistically significant differences in age or BMI among the study groups. The mean age was 35.75±7.23 years in the control group and 33.49±4.86 years in the azoospermic group, while the mean BMI was 21.35±1.61kg/m^2^ in the control group and 21.38±2.04kg/m^2^ in the azoospermic group. The mean sperm volume in the control group was 4.3±1.36 mL, which was significantly higher than 2.89±0.76 mL in the azoospermic group (p<0.05). Other infertile groups also showed reduced sperm volumes compared to the control, although these differences did not reach statistical significance.

**Table 1:** Comparison of patients’ characteristics, semen analysis between the study groups

Groups Variable	OAT (N=10)	OT (N=10)	OA (N=10)	Azoospermia (N=50)	Normospermia (N=20)	*p*-Value
Age (y)^a^	35.15±8.43	34.69±5.58	33±4.50	33.49±4.86	35.75±7/23	ns
Body mass index (kg/m^2^)^b^	21.19±2.05	20.56±2.09	22.19±1.20	21.38±2.04	21.35±1.61	ns
Sperm volume (ml)^b^	4.29±1.94	4.26±1.45	4.48±0.91	2.89±0.76^***^	4.3±1.36	<0.01
Sperm count (×10^6^/mL)^b^	7.76±4.58^***^	10.38±3.84^***^	7.42±4.03^***^	0^***^	73.25±25/34	<0.001
Progressive motility (%)^b^	12.85±10.38^***^	32.62±5^*^	17.14±1025^***^	0^***^	39.65±5.54	<0.001
Non-progressive motilit (%)^b^	7.69±3.09^**^	12.08±3.96	8.14±3.23^*^	0^**^	11.35±2.6	<0.001
Immotile sperm (%)^b^	79.46±11.47^***^	56.07±4.42^*^	74.71±10.55^***^	0^***^	45.5±4.38	<0.001
Morphology (%)^a^	1.6±0.51^*^	1.61±0.65^*^	4.71±0.55	0^*^	4.15±0.36	<0.001

Values are presented as mean ± SD The Kruskal-Wallis H (^a^) and One-way ANOVA (^b^) tests were used to determine if there are statistically significant differences between all groups and Normospermia group based on the distribution of all groups. OAT, Oligoasthenoteratozoospermia; OA, Oligoasthenospermia; OT, oligoteratozoospermia,

* *p*<0.05,

** *p*<0.01 and

*** *p*<0.001 were considered as significant values.

The mean sperm count in the control group was 73.25±25.34×10^6^/mL, whereas the azoospermic group showed an absence of sperm (p<0.001). Similarly, the OA, OT, and OAT groups had significantly lower sperm counts compared to the control. Progressive motility was 39.65±5.54% in the control group, significantly higher than 0% in the azoospermic group (p<0.001), with other infertile groups also showing significantly reduced progressive motility compared to the control. Non-progressive motility was 11.35±2.6% in the control group and 0% in the azoospermic group, indicating a significant difference (p<0.001), with other infertile groups similarly showing significantly lower non-progressive motility.

The percentage of immotile sperm was 45.5±4.38% in the control group, while it was 0% in the azoospermic group and significantly higher in the other infertile groups (p<0.001). Additionally, the mean percentage of normal sperm morphology was 4.15±0.36% in the control group and 0% in the azoospermic group, with the OA, OT, and OAT groups also exhibiting significantly lower normal morphology percentages compared to the control (p<0.001).

### qPCR results

**Figure 1:**
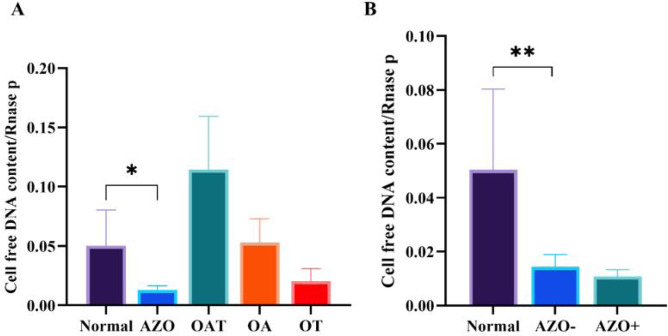
A and B: Comparison of cell-free DNA content in seminal plasma between control and different infertile groups. AZO: non-obstructive azoospermia, OAT:Oligoasthenoteratozoospermia, OA: Oligoasthenospermia and OT: Oligoteratozoospermia. AZO- : negative sperm retrieval, and AZO+ : positive sperm retrieval. Data are presented as mean ± SEM. *p<0.05 and **p<0.01 were considered as significant values.

In this study, significant differences in cfDNA content were observed between the control and experimental groups. The mean cfDNA content in the control group was 0.05±0.02, while in the azoospermic group, it was significantly lower at 0.01±0.003 (p=0.01). Additionally, the OAT group showed a higher cfDNA content with a mean of 0.11±0.04. Among other experimental groups, the OA group, with a cfDNA content of 0.05±0.02, and the OT group, with 0.02±0.01. When comparing cfDNA content between groups with and without sperm retrieval, the group with positive sperm retrieval (AZO+) had a cfDNA content of 0.01±0.002, while the group with negative sperm retrieval (AZO-) showed a higher cfDNA content of 0.01±0.004 (p=0.69) (Figure 1 and Table 2).

**Table 2:** Comparison of cell free DNA content between experimental groups and control

Groups	Experimental groups					Control group	*p*-Value
Cell free DNA content	OATa (N=10)	OTb (N=10)	OAc (N=10)	Azoospermiad (N=50)		Normospermiae (N=20)	
0.11±0.04 (10-3-0.4)	0.02±0.01 (10-4-0.14)	0.05±0.02 (10-4-0.15)	0.01±0.003 (10-4-0.09)		0.05±0.02 (0.005-0.4)	^a,e:^ 0.12 ^b,e:^ 0.17 ^c,e:^ 0.51 ^d,e:^ 0.01 ^f,e:^ 0.01 ^g,e:^ 0.58 ^f,g:^ 0.69
			Negative sperm retrievalf	Positive sperm retrievalg		
			0.01±0.004 (10-4-0.09)	0.01±0.002 (10-3-0.03)		

Samples included in this study were non-normally distributed (non-parametric) according to the values of the skewedness test, Kurtosis test, Z-value and Shapiro test. Data were presented as Mean ± SEM and min-max. The Kruskal-Wallis H test was used to determine if there are statistically significant differences between all groups and Mann-Whitney test was used to compare between two groups. OAT, Oligoasthenoteratozoospermia; OA, Oligoasthenospermia; OT, oligoteratozoospermia, *p*<0.05 was considered as significant values.

### Antioxidant and oxidative stress parameters

**Figure 2:**
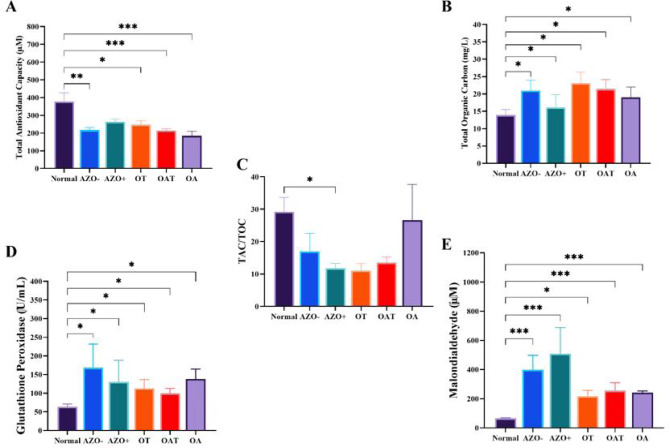
Comparison of Total Oxidative Stress (TOS) (A), Total Antioxidant Capacity (TAC) (B), TAC/TOC ratio (C), Glutathione Peroxidase (GPx) activity (D), and Malondialdehyde (MDA) levels (E) among different study groups. AZO- : non-obstructive azoospermia with negative sperm retrieval, AZO+:non-obstructive azoospermia with positive sperm retrieval OAT:Oligoasthenoteratozoospermia, OA:Oligoasthenospermia and OT:Oligoteratozoospermia.Data are shown as mean±SEM. Statistical significance is indicated by asterisks, with *p<0.05, **p<0.01, and ***p<0.001.

The examination of oxidative stress indicators and antioxidant defenses in seminal plasma among several study groups demonstrated noteworthy discrepancies, as illustrated in Figure 2. The mean Total Antioxidant Capacity (TAC) (Figure 2B) levels were notably lower in all infertile groups compared to the Normospermia group (p<0.01, p<0.05, p<0.001, respectively). Total Oxidative Stress (TOS) levels were significantly elevated in the infertile groups compared to the Normospermia (p<0.05) (Figure 2A). The TAC/TOS ratio was significantly lower in non-obstructive azoospermia with positive sperm retrieval groups compared to the Normospermia (p<0.05) (Figure 2C). Glutathione Peroxidase (GPx) activity was significantly higher in the infertile groups than in the control group (p<0.05) (Figure 2D). Additionally, Malondialdehyde (MDA) levels, were significantly higher in the infertile groups compared to the control (p<0.001, p<0.05) (Figure 2E).

## DISCUSSION

The findings of this study indicate a significant correlation between elevated cfDNA levels in semen and oxidative stress markers with compromised sperm parameters in infertile men, particularly among those with azoospermia and various forms of oligospermia. The complete absence of sperm volume and count in the azoospermic group, as well as the reduced counts observed in the OA, OT, and OAT groups, underscores the severity of reproductive challenges associated with these conditions. The substantial reduction in progressive motility in azoospermic individuals, along with decreased motility in the OA and OAT groups, highlights impaired sperm functionality as a key issue tied to oxidative damage. Furthermore, the findings reveal a marked decrease in sperm morphology across these groups, especially in azoospermic men, suggesting that oxidative stress may contribute to cellular damage, ultimately affecting sperm structure and quality. These results collectively emphasize the potential of cfDNA and oxidative stress markers as valuable biomarkers for assessing the severity of male infertility and provide insight into targeted therapeutic approaches to mitigate these effects. These findings highlight significant disparities in semen parameters, emphasizing the reproductive challenges faced by individuals with azoospermia, OA, OT, and OAT (Andrade et al., 2021; Shamohammadi et al., 2022).

The present study used a real-time PCR technique and an RNase P primer to quantify the levels of Cf-DNA in seminal plasma. Real-time PCR significantly reduces the danger of contamination, while amplifying the RNase P gene offers superior sensitivity and specificity compared to amplifying other genes like Actin or GAPDH The RNaseP DNA derived from the RNPP30 gene is highly abundant and exhibits reduced susceptibility to variability caused by the patient’s physiological condition (Romani et al., 2014).

Significant differences in cf-DNA levels in seminal fluid were found between infertile males and the control group. The AZO group had lower cf-DNA levels compared to the control group (p<0.01). The OA and OT groups had no significant differences. The AZO- group had lower cf-DNA compared to the Normospermia group (p<0.01). Our results align with studies that indicate a notable significance in CF-DNA levels in seminal plasma between patients with azoospermia and control patients. A study indicated a statistically significant difference (p=0.008) in Cf-DNA levels between patients with teratozoospermia and control subjects (Mbaye et al., 2021).

This suggests that poor spermiogenesis may be a relevant factor, as impaired sperm maturation triggers cellular checkpoints leading to cell death. This may explain the high free DNA levels in the teratozoospermia group. Additionally, azoospermia patients showed elevated seminal CF-DNA. To determine if this is linked to spermatozoa or germ cells, further pathological and biomolecular analysis is needed. (Tahmasbpour et al., 2014). The following hypothesis pertains to infections and inflammatory processes. The latter may be either particular or non-specific. Infection-induced inflammation can trigger cell lysis and phagocytosis, leading to the release of nucleic acids into the exosome. These nucleic acids are subsequently broken down by endonucleases and DNases. Alternatively, inflammation can arise from trauma, heat, or other local or systemic sources. An imbalance in the testicular environment might lead to necrosis and/or apoptosis of the support cells. The underlying mechanisms responsible for the excessive production of Cf-DNA have yet to be fully understood. 90% of seminal fluid is generated in glands that undergo substantial cell turnover. The prostate and seminal vesicles release circulating nucleic acids through active and/or passive means (Di Pizio et al., 2021; Gahan et al., 2008; Rykova et al., 2012; Stroun et al., 2001).

The examination of oxidative stress indicators and antioxidant defenses in seminal plasma among different research groups indicated that the total antioxidant capacity (TAC) was significantly reduced in all infertile groups compared to the control group (p<0.05). Conversely, the infertile groups had significantly elevated TOS levels compared to the control group (p<0.05). In addition, the activity of GPX was markedly elevated in all infertile groups as compared to the control group (p<0.05). Similarly, the concentrations of MDA were considerably elevated in the infertile groups compared to the control group (p<0.05). The results emphasize the increased oxidative stress and weakened antioxidant defenses in people with infertility, indicating that oxidative stress plays a crucial role in the development of male infertility (Agarwal & Prabakaran, 2005). Various investigations have shown that oxidative stress interferes with the antioxidant defense mechanism in seminal plasma, resulting in reduced antioxidant capacity and heightened vulnerability to oxidative damage (Kaltsas, 2023; Solorzano Vazquez et al., 2022; Walke et al., 2023). The results from various researches consistently support the idea that oxidative stress is responsible for causing harm to sperm, resulting in decreased sperm count and poor sperm function. Moreover, the disturbance of the antioxidant defense system worsens these consequences, hence leading to infertility as a whole (Baszyński et al., 2022; Evans et al., 2021). Increased levels of MDA were consistently detected in all groups of individuals experiencing infertility. This suggests heightened lipid degradation caused by oxidative stress. In addition, the level of GPX, a crucial antioxidant enzyme, was notably elevated in males who were unable to conceive. The observed rise in elevation is most likely a compensatory mechanism in response to elevated oxidative stress, as the body endeavors to mitigate the heightened amounts of ROS (Lanzafame et al., 2009; Qamar et al., 2023). Considering the significant impact of oxidative stress on male infertility, it may be advantageous to implement therapeutic approaches that focus on improving antioxidant defenses. Antioxidant supplementation has been suggested as a possible strategy to reduce oxidative damage and enhance semen quality.

This study investigated the varying importance of amounts of CF-DNA in semen, oxidative stress, and sperm characteristics in infertile men with non-obstructive and obstructive azoospermia. It emphasizes the crucial role of oxidative stress in male infertility. Although the groups had similar ages and BMIs, there were notable variations in semen parameters and oxidative stress indicators. The azoospermic group exhibited a total absence of sperm volume and count, while the OA, OT, and OAT groups displayed considerably reduced sperm counts and motility compared to the control group. Higher quantities of CF-DNA in the AZO and OAT groups indicate a greater extent of cellular damage. In addition, all groups experiencing infertility showed decreased TAC and increased TOS, which suggests an increased level of oxidative stress. Increased levels of GPX and MDA provided additional evidence of oxidative damage. The results emphasize the significance of oxidative stress in male infertility, indicating that antioxidant therapy may enhance the quality of semen. Further investigation is warranted to examine the efficacy of antioxidant therapies in reinstating fertility in men with infertility, to enhance therapeutic care and improve outcomes.

One limitation of this study is that statistical analyses were conducted without adjustments for potential confounding variables, such as age and BMI. This could have influenced the observed outcomes, as these factors may impact oxidative stress and cell-free DNA levels independently of infertility status. Future studies should incorporate adjustments for confounding variables to provide more accurate interpretations of the relationships between oxidative stress, cell-free DNA levels, and infertility. Another limitation is the imbalance in sample size between the control and patient groups, which may restrict comparison. Future studies should consider sample size calculations and age and BMI matching to improve result reliability.

## CONCLUSION

This study highlights the significant correlation between elevated cfDNA levels, oxidative stress, and impaired sperm parameters in men with azoospermia and oligospermia. The observed differences in cfDNA, antioxidant capacity, and oxidative damage between infertile groups and the normospermic control group suggest that cfDNA and oxidative stress markers are valuable indicators of sperm health and may serve as practical biomarkers for infertility assessment. These findings underscore the potential for cfDNA measurement and oxidative stress evaluation as diagnostic tools to classify the severity of infertility and guide treatment options. Moreover, our results support the consideration of antioxidant therapy as a potential strategy to reduce oxidative damage, enhance sperm quality, and improve reproductive outcomes for affected individuals.
